# Trace Elements in *Stenella coeruleoalba*: Assessment of Marine Environmental Pollution and Dolphin Health Status

**DOI:** 10.3390/ani14111514

**Published:** 2024-05-21

**Authors:** Clara Naccari, Vincenzo Ferrantelli, Gaetano Cammilleri, Stefano Ruga, Fabio Castagna, Roberto Bava, Ernesto Palma

**Affiliations:** 1 Department of Health Sciences, University “Magna Græcia” of Catanzaro, 88100 Catanzaro, Italy; rugast@gmail.com (S.R.); castagnafabio@yahoo.it (F.C.); roberto.bava@unicz.it (R.B.); palma@unicz.it (E.P.); 2 Istituto Zooprofilattico Sperimentale della Sicilia “A. Mirri”, 90129 Palermo, Italy; vincenzo.ferrantelli@izssicilia.it (V.F.); gaetano.cammilleri86@gmail.com (G.C.); 3 Interdepartmental Service Center—Center for Pharmacological Research, Food Safety, High Tech and Health (CIS-IRC–FSH) University “Magna Græcia” of Catanzaro, 88100 Catanzaro, Italy

**Keywords:** toxic and essential metals, *Stenella coeruleoalba*, marine environmental pollution, dolphins’ health status

## Abstract

**Simple Summary:**

Metals are ubiquitous environmental contaminants that can be easily accumulated and biomagnified in various fishes and mammalian species at the top of the aquatic food chain. Among marine mammalians, the striped dolphin (*Stenella coeruleoalba*) is considered to be a sentinel species of marine environmental pollution. The aim of this study was to assess, through Inductively Coupled Plasma-Mass Spectrometry (ICP-MS) analysis, the concentrations of toxic metals and metalloids, essential micro- and macro-elements in organs/tissues of *Stenella coeruleoalba*. Considering the low content of toxic metals and metalloids found, the analysis of some specific parameters was carried out: the marine pollution index (MPI) underlines the key role of dolphins to assess marine pollution, while the coefficient of condition (K) and the complete mineral profile are predictive of dolphins good health status. However, the correlations among toxic and essential metals, expressed as molar ratios, have shown that toxic metals cannot be detoxified by the analyzed essential metals.

**Abstract:**

Heavy metals are environmental contaminants and can easily accumulate and biomagnify in various marine species (fishes and mammalians) at the top of the aquatic food chain. Among marine mammalians, the striped dolphin (*Stenella coeruleoalba*) is the most abundant cetacean in the Mediterranean Sea and is considered to be a sentinel species to monitor the environmental marine pollution. In this study, the contents of toxic metals and metalloids (Cd, Pb, Hg, and As), micro-elements (Ni, Cr, Cu, Fe, Co, Mn, Se and Zn) and macro-elements (Na, Ca, K, Mg and P) were evaluated by ICP-MS analysis in several organs/tissues (lung, skin, muscle and liver) of *Stenella coeruleoalba*. The assessment of marine environmental pollution and dolphins health status was carried out through further analysis of the same specific parameters such as the metal pollution index (MPI) and coefficient of condition (K). Finally, the correlation between toxic metals and metalloids and essential micro-elements, expressed as molar ratios, was analyzed to evaluate the detoxifying ability (effectiveness) of Zn, Se and Cu. Data obtained showed the presence of toxic metals and metalloids analyzed in the *Stenella coeruleoalba* samples but the MPI values suggested a low environmental contamination of the Mediterranean Sea where dolphins lived. The content of micro- and macro-elements was found to be in a normal range for this species and predictive of dolphins good health status, as confirmed by the coefficient of condition K. However, the correlation between toxic and essential metals, expressed as molar ratios, showed that the following toxic metals cannot be detoxified by the essential metals: ^66^Zn/^201^Hg, ^82^Se/^201^Hg, ^63^Cu/^201^Hg and ^66^Zn/^52^Cr, ^82^Se/^52^Cr, ^63^Cu/^52^Cr. Therefore, this study highlights the key role of dolphin *Stenella coeruleoalba* to assess marine pollution and the importance of analyzing the complete mineral profile to evaluate the animal health status.

## 1. Introduction

Heavy metals are persistent environmental contaminants present in the aquatic ecosystem and assimilated by biota, and can be easily accumulated and biomagnified in predatory fishes and marine mammalians at the top of the aquatic food chain [1]. The quality of marine ecosystem is strongly influenced by the anthropogenic activity (fossil fuels combustion, exhaust gases, waste incineration, industrial waste, agricultural practices, aquaculture discharges, oil waste in the sea from tank washing, etc.) and natural process (volcanic activity, dust deposition, terrestrial crust erosion, etc.), which are responsible for metals release and significantly affect the health of both the marine environment and animal species. In fact, these environmental pollutants could cause alterations in marine habitats and compromise the biodiversity of species living in this ecosystem (fishes, birds, algae, etc.) [2,3]. The presence of metals in marine environment, however, is also influenced by pH, temperature, sea composition, microorganisms, redox conditions, ability of detoxifying systems, integrity of excretion routes, etc. [2]; therefore, the accumulation of metals in marine species, used as bioindicators, reflects the contamination of the marine environment where they lived [4,5,6].

In the last decades [7,8,9], several eco-toxicological studies focused their attention on the pollution in the Mediterranean Sea and its effect on the marine species and the peculiar characteristics of this area. The Mediterranean Sea, in fact, is a basin with a high population density, maritime traffic and urbanization of the coastlines, particularly in the summer period, and for this reason it is highly exposed to the circulation of pollutants such as heavy metals [10]. Due to its geo-morphological characteristic and the presence of submarine volcanoes, the sea bottom is naturally rich in trace elements, particularly Hg, and through chemical and biochemical processes, toxic compounds such as methylmercury can be released and enter the aquatic food chain through plankton. In addition, its natural structure of semi-enclosed basin promotes the bioconcentration of these pollutants and the subsequent biomagnifications in marine species according to the trophic levels. Therefore, the pollution of Mediterranean Sea strongly affects the marine environment, aquatic wildlife and quality of fishery products.

Among marine mammalians, dolphins can serve as sentinel species to monitor the environmental pollution [11], according to the Marine Strategy Framework Directive (MSFD) [12], because they are long-lived, predatory, reside in coastal areas, and the metals exposure in these species is an expression of danger for the aquatic ecosystem, which is useful for risk assessment of marine environmental pollution [13]. This species can accumulate pollutants through several routes (ingestion of food, sea water, breathing, skin, placenta, etc.) [1,14,15]. Metals exposure in these marine mammalians is influenced by several factors such as habitat, food habits, physio-pathological status of the animal, age, and sex [16]. Being an organism at the top of the aquatic food chain, dolphins consume mainly pelagic, cephalopod and demersal fishes [17,18,19,20], which are a source of essential minerals, such as Se, Zn, Cu, macronutrients, protein, lipids and vitamins, but at the same time, they introduce toxic metals, particularly Hg and Pb, which are responsible for adverse effects at low concentrations. As documented by our previous studies [21] and by other authors [22,23], pelagic and cephalopod fishes are good accumulators of heavy metals and they are adsorbed according to the trophic transfer factor, reaching very abundant concentrations in predatory fishes [6] and, particularly, in marine mammalians [4]. Toxic metals, in fact, could affect several biological functions and cause neurotoxicity, immune-suppression, endocrine disruption, and susceptibility to infections from pathogens, etc. [2,24,25,26], and can be responsible for multiple symptomatic effects that are capable of compromising their health and survival. In addition, toxic metals may influence the growth, metabolism, nutrition state, and maturity of young individuals [16,27], reducing the bioavailability of essential minerals and substitution in biological processes [28]. However, the marine mammalians activate several processes to counteract the toxic effect of metals and prevent their accumulation in organs and tissues according to metal affinity [29] such as storage, biotransformation, excretion, homeostatic regulation, release of metallothioneines, and detoxification processes. Therefore, the study of complete mineral profile is useful to assess dolphins’ good health status.

The aim of this study was to evaluate the content of toxic metals and metalloids as well as essential micro- and macro-elements in organs/tissues of striped dolphin (*Stenella coeruleoalba*), the most abundant cetacean present in the Mediterranean Sea and to assess both the marine environmental pollution and dolphins’ health status. Finally, the correlation between toxic and essential metals, expressed through the analysis of molar ratios of each element, was evaluated to know the ability (effectiveness) of the detoxifying system in this marine mammalian.

## 2. Materials and Methods

### 2.1. Reagents

Ultrapure water (resistivity of 18 MΩ cm), HNO_3_ (70% *v*/*v*), and H_2_O_2_ (30% *v*/*v*) for trace metal analysis were purchased from J.T. Backer (Mallinckrodt Backer, Milan, Italy). The stock standard solutions (1000 mg L^−1^ in 2% nitric acid) of each element and online internal standards of Sc, Ge, In, and Bi (1000mg L^−1^ in 2% nitric acid) were purchased from Fluka (Milan, Italy).

### 2.2. Sampling

**Figure 1:**
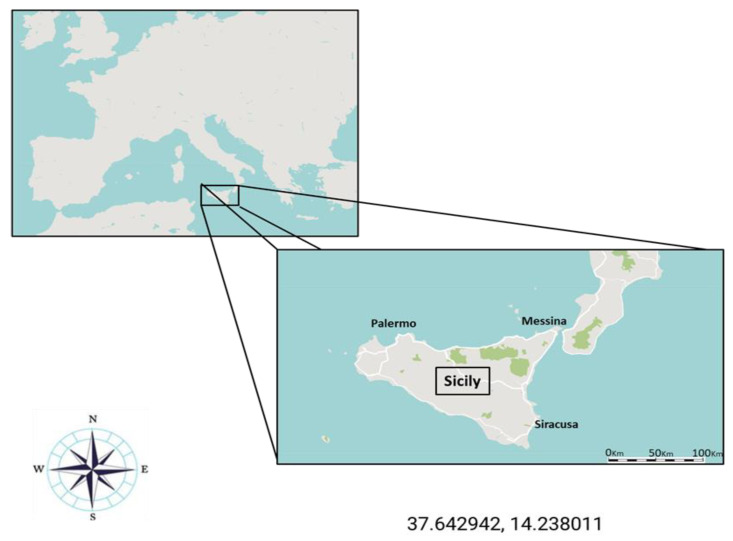
Collection site of dolphins stranded in the Sicilian coast of the Mediterranean Sea.

Striped dolphins (*Stenella coeruleoalba*) were collected dead by the Istituto Zooprofilattico Sperimentale della Sicilia “A. Mirri”, Palermo (Italy). All samples were stranded along the Sicilian coast of the Mediterranean Sea (Figure 1), with different states of conservation of carcasses, reported according to the National score (Table 1).

The necropsies of dolphins showed the absence of particular signs on the carcass, no evidence of bacteria from the microbiological analysis, and not useful data to identify the causes of death. The animals were of different sex, weight, length, and age. In relation to the age, the length was used as an index of the different development stages. More precisely, a length between 0.95 and 1.80 m was considered to be indicative of juveniles, while >1.81 m was indicative of adults [30]. From each dolphin, samples of liver, muscle, lung and skin were taken and preserved in PET containers, and finally frozen at −20 °C until analysis.

### 2.3. Sample Preparation

All organs/tissues of each dolphin were homogenized, and then mineralized according to the method by Naccari et al. 2015 [21] and Ferrantelli et al. 2012 [5]. Briefly, all samples (0.5 g), previously homogenized, were digested with HNO_3_ (70%) and H_2_O_2_ (30%) in a closed-vessel microwave digestion system (CEM Microwave^TM^ Digestion System, Discovery SP-D, CEM Corporation, Mathews, NC, USA), and finally submitted to analysis in ICP-MS for metals determination. Each set of samples was analyzed in the presence of a blank and processed in a similar manner. All determinations were carried out in triplicate. All glassware used for the analysis was treated with HNO_3_ (15%) overnight to avoid contamination, then rinsed with ultrapure water and dried prior to use.

### 2.4. Analysis in Inductively Coupled Plasma-Mass Spectometry (ICP-MS)

The metals analysis has been carried out using an ICP-MS spectrometer equipped with an auto-sampler ASX520 (Cetac Technologies Inc., Omaha, NE, USA) under the following conditions: RF power, 1550 W; plasma gas flow rate, 14 L min^−1^; auxiliary gas flow rate, 0.89 L min^−1^; carrier gas flow rate, 0.91 L min^−1^; helium collision gas flow rate, 4.5 mL min^−1^; spray chamber temperature, 2.70 °C; sample depth, 4.27 mm; sample introduction flow, 0.93 mL min^−1^; nebulizer pump, 0.1 rps; extract lens 1 voltage, 1.5 V. The instrument has been operated in He KED mode to remove spectral interferences for the low and high mass elements using the following monitored isotopes: ^111^Cd, ^208^Pb, ^202^Hg, ^75^As, ^60^Ni,^52^Cr,^57^Fe, ^66^Zn,^63^Cu, ^82^Se, ^59^Co, ^55^Mn,^23^Na, ^39^K, ^44^Ca,^24^Mg, ^31^P, and online internal standards: ^45^Sc, ^72^Ge, ^209^Bi, ^115^In.

### 2.5. Validation Method

In Table 2, all analytical parameters of the validation method were reported. The accuracy was assessed by the analysis of certified reference material DOLT-5 (dogfish liver reference material for trace metals) from the National Research Council of Canada; the precision was expressed as the relative standard deviation (RSD%) of four independent determinations; the specificity was confirmed by the analysis of blanks to exclude contamination and interference during the analysis and to confirm reagents purity. Good laboratory practice (GLP) was applied throughout and procedural blanks were analyzed.

### 2.6. Assessment of Metals Pollution and Dolphin Health Status

The risk of metals marine pollution was assessed using a specific parameter such as the metal pollution index (MPI), which calculates the metals accumulation levels in each organ and tissue [31,32] according to the following formula:

MPI = (M1 × M2 × M3 × … Mn)/n(1)

where Mn is the concentration of “n” metal (mg/kg) in a specific tissue sample. According to Jamil et al. 2014 [33], values of MPI among the range of 5–10 express a low contamination, among the range of 2–5 very low contamination, and values <2 express a not significant contamination. 

The dolphin’s health status, instead, was evaluated through the coefficient of condition (K) (Figure 1). The parameters used to express the relationship between weight and length [31,34] were calculated for each sample according to the following Fulton equation:

K = 100 × W/L^3^ (2)

where W is the weight (g) and L is the body length (cm), with a value of 1 considered as a safety level.

**Figure 2:**
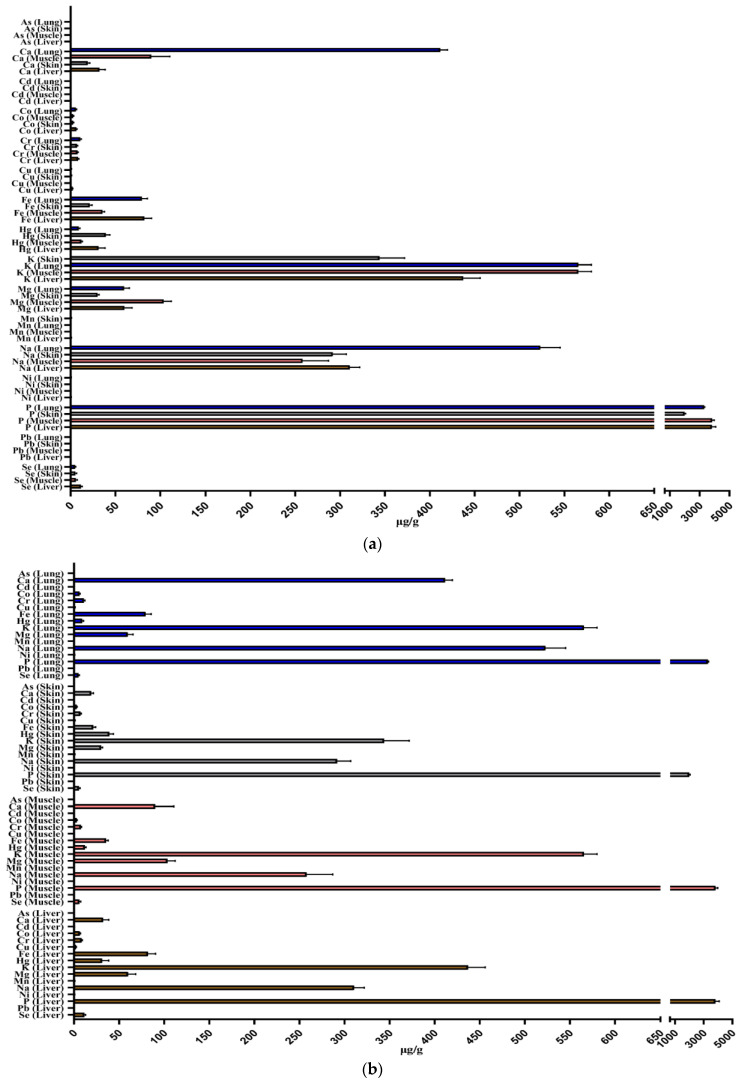
(**a**,**b**) Statistical distribution of each metal analyzed (MV ± SD µg g^−1^) in different organs/tissues of *Stenella coeruleoalba* samples.

In addition, the length–weight relationship was evaluated through a linear regression analysis (Figure 2) and expressed according to the following equation:

logW = log *a* + *b* logL (3)

where *a* is the intercept and *b* is the slope.

The correlation among toxic and essential metals has been expressed as molar ratios, which is calculated using the atomic mass of each element: ^111^Cd, ^208^Pb, ^201^Hg, ^75^As, ^52^Cr, ^60^Ni, ^66^Zn, ^82^Se, ^63^Cu. As described by Méndez-Fernandez et al. 2014 [35], values corresponding to 1 were considered as protection index.

### 2.7. Statistical Analyses

The statistical analyses for this study were computed in Microsoft Excel and GraphPad PRISM (version 9.0, GraphPad program Inc., La Jolla, CA, USA). Shapiro-Wilk test for normality and Barlett’s test for homogeneity were performed. The Kruskal-Wallis test was used to compare the concentration of all metals analyzed in various organs/tissues. Data are expressed as mean values ± SD of at least three determinations. The differences were considered statistically significant when the *p*-value was <0.05. The linear regression analysis was used to evaluate the relationship between variables considered in the study.

## 3. Results 

The results showed the presence of all metals analyzed in striped dolphin samples (Table 3, Table 4 and Table 5). From the statistical analysis carried out to correlate the distribution of each metal, significant differences were observed (Figure 2a). Hg concentrations were higher in skin (41.51 ± 2.15 µg g^−1^) and liver (31.22 ± 1.24 µg g^−1^) than in the lung and muscle (*p* < 0.001). Additionally, concentrations in all different organs/tissues analyzed were among the lowest and statistically not different from each other metal. Cd, as well as Pb, Ni, Cr, Co, Mn, Cu, Zn, Se, and P, showed no significant differences in their concentrations in different organs/tissues. Fe was found to be statistically more concentrated in lung (79.53 ± 2.18 µg g^−1^) than in skin (*p* < 0.001) and muscle (*p* < 0.001), but not compared to the liver, which presented comparable concentrations (82.24 ± 1.18 µg g^−1^). Ca was statistically more concentrated in the lung (411.32 ± 37.29 µg g^−1^) than in the skin (*p* < 0.001), muscle (*p* < 0.05), and liver (*p* < 0.001). K present in the lung (388.23 ± 43.30 µg g^−1^) was statistically higher than in the skin (*p* < 0.001) and liver (*p* < 0.01), but not statistically different than in the muscle. Mg was statistically more concentrated in muscle (103.87 ± 32.16 µg g^−1^) compared with liver (*p* < 0.05) and with skin (*p* < 0.001), but in concentrations not statistically significant when compared with the lung. Na concentrations were higher in the lung (534.54 ± 42.35 µg g^−1^) than in the skin, muscle, and liver (*p* < 0.001). Considering, instead, the distribution of all metals in organs/tissues analyzed, it is possible to observe the following differences (Figure 2b). In the lung, the most abundant element was P (*p* < 0.001) with a mean concentration of 3296.55 ± 51.28 µg g^−1^, followed by K and Na (388.23 ± 43.30 µg g^−1^ and 534.54 ± 42.35 µg g^−1^, respectively). In the skin, P was also the most abundant element (1989.84 ± 38.25 µg g^−1^) compared with all other elements (*p* < 0.001); K and Na, reaching an average concentration of 344.03 ± 35.29 µg g^−1^ and 291.89 ± 23.29 µg g^−1^, respectively, were similarly significant after P compared with the other elements analyzed (*p* < 0.001). In muscles, P reported an average concentration of 3834.72 ± 54.27 µg g^−1^, proving to be the most abundant element among all those examined (*p* < 0.001), followed by K (568.09 ± 21.28 µg g^−1^) and Na (284.0 ± 41.08 µg g^−1^). The same pattern of P in the organs just discussed was shown in the liver, thus being the statistically most concentrated element (3824.01 ± 31.29 µg g^−1^). K (437.33 ± 25.38 µg g^−1^) and Na (311.07 ± 35.09 µg g^−1^) replicated the same trend observed in the different organs, including liver, being its highest concentrations after those of P.

No significant differences were found among metals content according to the different geographical areas where the dolphin samples were collected.

In Table 3, the MPI is reported. This parameter, calculated on concentrations of toxic metals and metalloids in all organs/tissues of dolphins, with greater interest for liver (0.3616), showed very low values (MPI < 2) in all samples.

**Figure 3:**
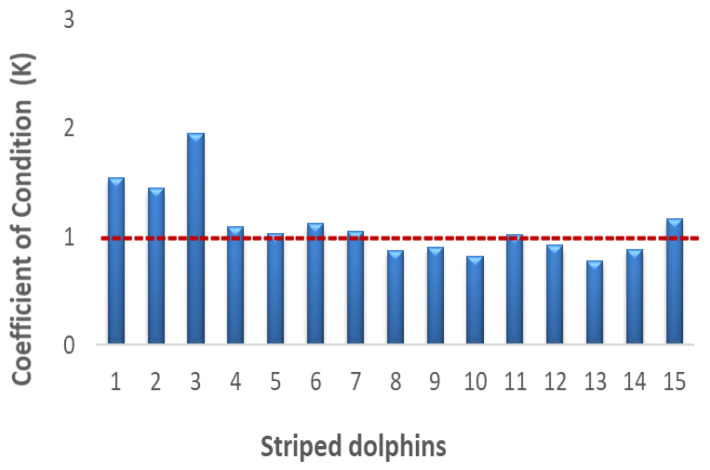
Coefficient of condition (*K*) in *Stenella coeruleoalba* samples. The red line corresponds to the safety level 1.

Data relating to the coefficient of condition (K) are reported in Figure 3 and showed high values in all dolphin samples around or > 1 (value considered as safety level).

**Figure 4:**
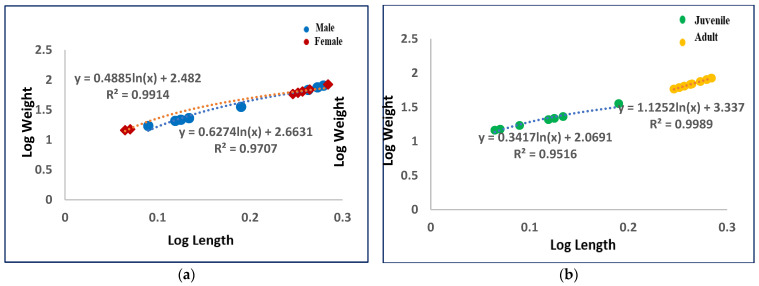
Length–weight relationship in (**a**) male and female and (**b**) juvenile and adult samples of *Stenella coeruleoalba*.

A further analysis of length–weight relationship of dolphin samples was carried out to evaluate the animal welfare and relative growth. It showed a positive linear regression according to the sex (Figure 4a) and the development stage (age) (Figure 4b) of dolphins.

Considering the content of all mineral elements analyzed in striped dolphin samples, the correlation among toxic metals and metalloids and essential micro-elements, expressed as molar ratios, is reported in Table 6. Particularly, the molar ratios ^66^Zn/^201^Hg, ^82^Se/^201^Hg, ^63^Cu/^201^Hg and ^66^Zn/^52^Cr, ^82^Se/^52^Cr, ^63^Cu/^52^Cr in all organs/tissues analyzed were <1 (protection index), depending on Hg levels in the denominator [20,36]. 

## 4. Discussion

The metals content found in dolphin samples is correlated to the specific affinity for different organs and tissues. Metals in liver are due to storage, sequestration, detoxification processes, and homeostatic regulation; the lung and skin represent an important route of adsorption/elimination of metals in marine environment; the content in muscle is indicative of chronic exposure according to the diet [2,15]. 

The analysis of toxic metals found in organs/tissues of *Stenella coeruleoalba* put in evidence the highest Hg levels, particularly in the skin and liver, and this could be attributed to the marine pollution of habitat where dolphins were living and feeding.

The presence of Hg and all trace metals found in dolphin samples is clearly linked to the mineral composition of Mediterranean Sea bottom and its natural structure of semi-enclosed basin where pollutants could be easily concentrated. However, considering the MPI, a specific parameter of risk assessment which reflects the diffusion of metals from the aquatic environment into different organs and tissues of marine species [37,38], data obtained with value <1 (safety levels) in all tissues analyzed were indicative of a low pollution level of Mediterranean Sea, suggesting a not significant contamination of marine environment where dolphins lived. Although this index has been proposed for different matrices, such as waters, sediments and in filter-feeding organisms, it is actually applied in a study on several varieties of fishes [39,40] to correlate the impact of environmental pollution on these species, that are living in contact with the sea-bottom and intermediate waters. However, in predatory fishes and marine mammalians, MPI could contribute to providing information on metal distribution, which is introduced according to feeding habits through the trophic levels until the top of the aquatic food chain. 

The study of all essential elements is important to evaluate the health status of animals [41] or if their accumulation/lack is associated with disorders and pathologic conditions that are able to influence the normal growth. It is known that the micro-elements, such as Fe, Cu, Zn, Se, play several essential functions in the body as constituents of soft tissues and biological fluids and are important cofactors of enzymatic systems, but can become toxic at higher levels. The macro-elements, such as K, Na, Mg and Ca, are needed for the hydro-electrolytic balance, cellular electric potential, and homeostasis; Na and K are needed in the regulation of osmotic pressure; P in energy production, Mg in fat and protein synthesis; Ca and P in bone structure [42,43]. 

The studies on macro-elements in marine mammalians are few [44] and generally focused on their content in liver for its storage, regulation and detoxifying processes [45]. The present study, instead, reports that the mineral content in the skin tissue is metabolically active in marine mammalians [46], where the essential metals are important to guarantee the epidermic structure and avoid disorders, whereas in muscle and lung, these elements are responsible for muscle performance and breathing, respectively.

In general, our results on the content of micro- and macro-elements found in dolphin samples were in a normal range for *Stenella* spp., in comparison with data reported in literature by other authors [2] and not evidence of conditions of accumulation/lack; therefore, this could be considered predictive of dolphins’ good health status. 

The coefficient of condition, which is a biometric parameter used to assess both the habitat quality and marine species health status, provided useful information of the organism’s development [47], according to the specific energy level, physiological or pathological status of animal, etc. The values >1 found for all dolphins analyzed are an expression of a balanced feeding and indicate that the *Stenella coeruleoalba* samples of this study were in good health status. Also, the length–weight relationship, influenced by several factors such as habitat, fish activities, feeding habits, seasons, temperature, etc. [48,49], demonstrated the animal welfare and regular growth of dolphin samples, with a positive linear regression according to the sex and the development stage (age). Therefore, these specific parameters confirmed the health status of our samples of *Stenella coeruleoalba* from the Mediterranean Sea.

In addition, the correlation between toxic and essential metals, evaluated through their molar ratios, was considered to better understand the health status of animals and the effects of exposure to these pollutants on their organism. It is known, in fact, that the essential metals (Se, Zn and Cu) can protect the animals against heavy metals, particularly Hg, through antioxidant activity, competition for binding sites, and demethylation of methyl-Hg; however, the formation of Hg-Se could be responsible for a deficiency of free Se, while the Me-Hg could be inhibited by selenium enzymes [25,50,51,52,53]. In this study, the molar ratios obtained values <1 and showed that Hg and Cr cannot be detoxified by the essential metals Zn, Se, and Cu, which are present in enzymatic systems. This is probably due to the sequestration mechanism, deficiency of essential metals, etc.

## 5. Conclusions

This study showed that the presence of toxic metals and metalloids, particularly Hg, found in significant concentrations in all organs/tissues of striped dolphins is correlated to marine environmental pollution, although a not significant pollution level of Mediterranean Sea ecosystem was documented in this study from low MPI values. The exposure to toxic metals is directly influenced by the food habits of these marine mammalians, that were feeding mainly of pelagic, cephalopods and demersal fishes, which are considered good accumulators of heavy metals.

The content of micro- and macro-elements, introduced with the diet, documents the dolphin’s good health status, as confirmed by the coefficient of condition (K) calculated for each dolphin sample, although, as showed by molar ratios among the toxic and essential metals analyzed, the levels of detoxifying metals, Se, Zn and Cu, are unable to carry out a protective action against Hg and Cr, probably due to deficiency, sequestration or presence of other pollutants.

This study, therefore, underlines the key role of dolphins in eco-toxicological studies, as sentinel species to assess marine environmental pollution, especially in a very polluted coastline area with a high population density and maritime traffic, such as the Mediterranean basin, where aquatic species are subjected to a long-term exposure to contaminants. 

The analysis of complete mineral profile is demonstrated to be useful in the assessment of both marine pollutions and dolphins’ health status. In fact, trace metals were adsorbed and bio-accumulated in these marine mammalians according to the feeding habits, physiological or pathological state, and specific characteristics of animals, such as age and sex. 

In conclusion, the studies on metals exposure in *Stenella coeruleoalba* could be periodically carried out to guarantee the quality of its marine ecosystem and health status, considering the increased phenomena of dolphins’ stranding, which is observed more often in the last period in the Mediterranean coastlines.

## Figures and Tables

**Table 1:** Characteristics of striped dolphins (*Stenella coeruleoalba*) collected stranded along the Sicilian coast of the Mediterranean Sea.

Sample	Sex	Weight (kg)	Length (m)	Developmental Stage	Collection Site	^a^ State of Beached Carcasses
1	F	14.5	0.98	Juveniles	Siracusa	2
2	M	35.5	1.35	Juveniles	Messina	2
3	M	20.7	1.02	Juveniles	Palermo	2
4	M	17	1.16	Juveniles	Siracusa	2
5	M	21.6	1.28	Juveniles	Messina	3
6	F	15	1.10	Juveniles	Messina	2
7	M	23	1.30	Juveniles	Palermo	2
8	F	58	1.88	Adult	Siracusa	3
9	M	80	2.07	Adult	Siracusa	2
10	F	61	1.95	Adult	Messina	2
11	F	69	1.89	Adult	Palermo	3
12	F	64	1.91	Adult	Palermo	2
13	F	84	2.21	Adult	Messina	2
14	M	75	2.04	Adult	Siracusa	3
15	M	68	1.80	Adult	Siracusa	2

^a^ State of conservation of beached carcasses, expressed according to a score system: 1 alive/just deceased, 2 fresh carcass, 3 moderately decomposed carcass, 4 advanced decomposed carcass, 5 mummified carcass or skeletal remains.

**Table 2:** Parameters of the analytical method.

Element	Linearity (R^2^)	LOD ^a^ (ng/g)	LOQ ^b^ (ng/g)	DOLT-5 ^c^ (µg/g)	Observed Values (µg/g)	Recovery (Range and %)		RSD ^d^ (%)
Hg	0.997	0.022	0.042	0.44 ± 0.18	0.33 ± 0.08	97–102%	(99.8)	1.122
Pb	1.001	0.146	1.038	0.162 ± 0.032	0.154 ± 0.012	96–123%	(99.7)	0.418
Cd	0.998	0.022	0.031	14.5 ± 0.6	14.1 ± 0.43	98–134%	(99.9)	1.775
As	0.999	0.014	0.021	34.6 ± 2.4	33.8 ± 0.32	95–122%	(99.9)	1.273
Ni	0.991	0.041	0.184	1.71 ± 0.56	1.62 ± 0.53	92–133%	(98.6)	4.32
Cr	0.999	0.021	0.199	-	-	92–136%	(99.1)	1.410
Se	0.999	0.019	0.063	8.3 ± 1.8	7.75 ± 0.68	98–184%	(98.9)	1.454
Co	0.999	0.006	0.026	0.267 ± 0.026	0.19 ± 0.06	96–149%	(98.2)	1.048
Mn	0.996	0.104	0.162	105.3 ± 5.4	100.7 ± 6.72	95–118%	(99.3)	2.521
Cu	0.995	0.152	1.495	35.0 ± 2.4	32.22 ± 0.86	98–111%	(99.1)	2.753
Zn	0.990	0.012	0.721	105.3 ± 5.4	108.2 ± 8.04	97–110%	(99.5)	4.052
Fe	0.999	0.014	0.493	1070 ± 80	998 ± 13.2	97–111%	(98.1)	1.094
Na	0.998	0.181	1.722	9.900 ± 1600	9561± 87.4	98–110%	(99.7)	1.111
Ca	0.997	0.153	1.586	550 ± 80	508 ± 67	97–115%	(98.2)	1.542
K	0.999	0.168	1.778	14.400 ± 300	13.980 ± 450	98–118%	(99.1)	2.018
Mg	0.998	0.144	1.825	940 ± 100	912 ± 65	97–125%	(99.3)	1.756
P	0.997	0.211	1.967	-	-	98–132%	(99.1)	1.438

^a^ LOD: limit of detection. ^b^ LOQ: limit of quantification. ^c^ Dogfish liver certified reference material for trace metals and other constituents from the National Research Council of Canada (NRC-CNRC)**.** ^d^ RSD (%): relative standard deviation of five independent determinations.

**Table 3:** Levels of toxic metals and metalloids in organs/tissues of striped dolphin (*Stenella coeruleoalba*), expressed as µg g^−1^ of individual measurements and metal pollution index (MPI).

		Cd	Pb	Hg	As	MPI
	Mean	0.029	0.25	^c^ 9.29	0.12	
LUNG	S.D	0.01	0.01	1.62	0.008	0.0020
	Median	0.03	0.17	6.28	0.101	
	Mean	0.076	0.17	^c^ 41.51	0.31	
SKIN	S.D	0.004	0.006	2.15	0.007	0.0408
	Median	0.07	0.126	38.92	0.229	
	Mean	0.049	0.21	^a^ 12.11	0.16	
MUSCLE	S.D	0.07	0.05	1.02	0.004	0.0050
	Median	0.041	0.152	9.84	0.048	
	Mean	0.54	0.26	^c^ 31.22	0.33	
LIVER	S.D	0.03	0.01	1.24	0.08	0.3616
	Median	0.26	0.16	11.17	0.24	

^a^ *p* < 0.05, ^c^ *p* < 0.001 vs. other metals analyzed.

**Table 4:** Microelements in organs/tissues of striped dolphins (*Stenella coeruleoalba*), expressed as µg g^−1^ of individual measurements.

		Cr	Ni	Fe	Co	Mn	Se	Zn	Cu
	Mean	10.98	0.81	^c^ 79.53	6.03	0.42	5.14	8.14	1.09
LUNG	S.D	1.28	0.03	2.18	2.19	0.04	0.96	2.19	0.62
	Median	9.06	0.65	79.22	7.16	0.21	4.81	5.18	1.03
	Mean	7.27	0.47	21.45	3.02	0.98	5.48	6.67	1.16
SKIN	S.D	1.73	0.03	3.17	0.08	0.07	0.82	1.26	0.74
	Median	7.07	0.45	18.37	2.68	0.083	2.39	10.34	0.97
	Mean	7.86	0.54	35.63	3.03	0.14	6.05	7.25	1.09
MUSCLE	S.D	1.02	0.01	1.98	0.02	0.08	0.71	0.82	0.28
	Median	5.07	0.46	32.56	2.01	0.14	2.21	5.79	1.02
	Mean	8.54	0.77	^c^ 82.24	6.54	0.84	11.59	8.29	2.35
LIVER	S.D	1.02	0.03	1.18	1.27	0.02	1.92	0.5	0.81
	Median	6.59	0.52	64.05	8.04	0.61	8.50	10.96	2.73

^c^ *p* < 0.001 vs. other metals analyzed.

**Table 5:** Macro-elements in organs/tissues of striped dolphins (*Stenella coeruleoalba*) expressed as µg g^−1^ of individual measurements.

		Na	K	Ca	Mg	P
	Mean	534.54	388.23	411.32	59.78	^c^ 3296.55
LUNG	S.D	42.35	43.30	37.29	11.82	51.28
	Median	523.56	374.45	428.87	49.96	3311.01
	Mean	291.89	^c^ 344.03	^c^ 19.24	^c^ 30.21	^c^ 1989.84
SKIN	S.D	23.29	35.29	1.23	7.18	38.25
	Median	314.77	412.32	14.56	31.96	2075.93
	Mean	284.01	568.09	^a^ 90.13	103.87	^c^ 3834.72
MUSCLE	S.D	41.08	21.28	26.21	32.16	54.27
	Median	222.28	562.90	19.33	104.74	4036.19
	Mean	^c^ 311.07	^b^ 437.33	^c^ 32.38	^a^ 60.17	^c^ 3824.01
LIVER	S.D	35.09	25.38	9.27	21.19	31.29
	Median	298.17	426.82	18.62	30.68	3394.25

^a^ *p* < 0.05, ^b^ *p* < 0.01, ^c^ *p* < 0.001 vs. other metals analyzed.

**Table 6:** Molar ratio between toxic metals and essential micro-elements in organs/tissues of striped dolphins (*Stenella coeruleoalba*).

Ratio	LUNG	MUSCLE	LIVER	SKIN
^66^Zn/^201^Hg	0.19	0.59	0.26	0.13
^82^Se/^201^Hg	0.12	0.49	0.18	0.10
^63^Cu/^201^Hg	0.03	0.09	0.07	0.02
^66^Zn/^207^Pb	32.56	34.52	31.88	39.94
^82^Se/^207^Pb	20.55	28.80	21.58	32.81
^63^Cu/^207^Pb	4.38	5.22	9.03	6.97
^66^Zn/^112^Cd	281.87	95.62	147.95	87.76
^82^Se/^112^Cd	177.99	79.85	21.46	72.10
^63^Cu/^112^Cd	37.97	16.69	37.99	47.61
^66^Zn/^75^As	67.83	44.92	25.12	21.55
^82^Se/^75^As	42.72	37.48	16.94	17.67
^63^Cu/^75^As	9.14	6.78	7.11	3.75
^66^Zn/^60^Ni	45.22	44.18	42.12	14.14
^82^Se/^60^Ni	28.56	24.25	28.40	9.89
^63^Cu/^60^Ni	6.09	6.79	11.93	2.46
^66^Zn/^52^Cr	0.74	0.92	0.97	0.91
^82^Se/^52^Cr	0.46	0.77	0.65	0.75
^63^Cu/^52^Cr	0.02	0.14	0.27	0.16

## Data Availability

All data and results related to this study are included in the article.
